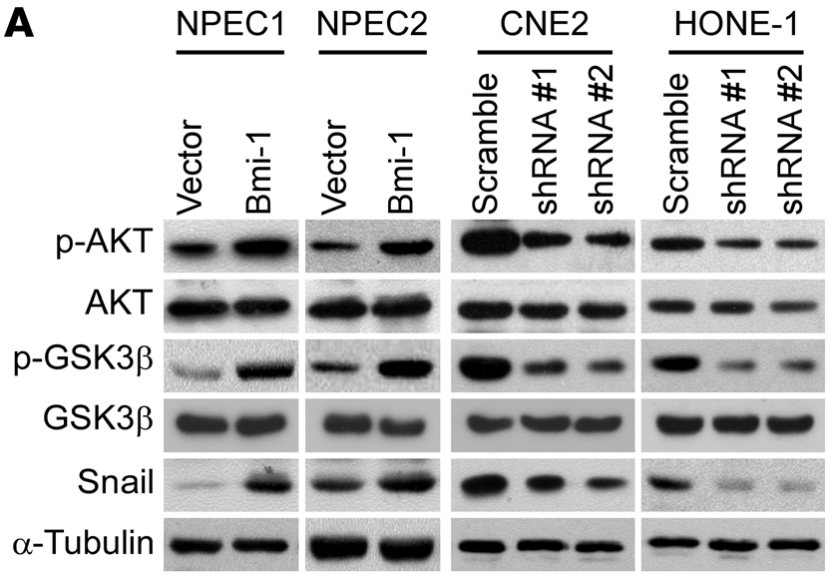# Corrigendum to The polycomb group protein Bmi-1 represses the tumor suppressor PTEN and induces epithelial-mesenchymal transition in human nasopharyngeal epithelial cells

**DOI:** 10.1172/JCI200436

**Published:** 2025-11-03

**Authors:** Li-Bing Song, Jun Li, Wen-Ting Liao, Yan Feng, Chun-Ping Yu, Li-Juan Hu, Qing-Li Kong, Li-Hua Xu, Xing Zhang, Wan-Li Liu, Man-Zhi Li, Ling Zhang, Tie-Bang Kang, Li-Wu Fu, Wen-Lin Huang, Yun-Fei Xia, Sai Wah Tsao, Mengfeng Li, Vimla Band, Hamid Band, Qing-Hua Shi, Yi-Xin Zeng, Mu-Sheng Zeng

Original citation: *J Clin Invest*. 2009;119(12):3626–3636. https://doi.org/10.1172/JCI39374

Citation for this corrigendum: *J Clin Invest*. 2025;135(21):e200436. https://doi.org/10.1172/JCI200436

The authors became aware that in Figure 3A, the GSK3β blot for the NPEC2 cells was inadvertently duplicated in the CNE2 panel. In addition, in Supplemental Figure 6A, the representative image for 24-hour Bmi1 shRNA#2 + PTEN shRNA#2 (row 3, column 4) was inadvertently duplicated from the representative image for 24-hour Bmi1 shRNA#2 + PTEN shRNA#1 (row 3, column 3). The correct Figure 3A is shown below and an updated version of the supplemental material has been provided.

The authors regret the errors.

## Supplementary Material

Supplemental data

## Figures and Tables

**Figure 3A:**