# Supplementary material for: Corrigendum to The polycomb group protein Bmi-1 represses the tumor suppressor PTEN and induces epithelial-mesenchymal transition in human nasopharyngeal epithelial cells
Source: J Clin Invest. 2025 Nov 3;135(21):e200436. doi: 10.1172/JCI200436 (PMC12578399; doi:10.1172/JCI200436)
Supplement: Supplemental data [file jci-135-200436-s317.pdf]

Supplemental Data

Supplemental Methods

Plasmids and antibodies. The pMSCV-Bmi-1 and Bmi-1 short hairpin RNA (shRNA) constructs were generated as described previously (1, 2). The pSuper-retro-constructs containing *PTEN* shRNA were generated by cloning the following *PTEN*-specific RNAi target sequences into pSuper-retro: PTEN shRNA #1: 5-GATCTTGACCAATGGCTAAGT-3; PTEN shRNA #2: 5-CGGGAAGACAAGTTCATGTACTT-3. Luciferase reporter gene constructs containing the E-cadherin promoter were cloned into the promoter-less pGL3 enhancer plasmid vector. The following antibodies were used: mouse anti-Bmi-1 (Upstate Biotechnology), mouse anti-E-cadherin, α-Catenin, Fibronectin and Vimentin (Transduction Laboratories), mouse anti-PTEN and rabbit anti-Snail (Santa Cruz Biotechnology Inc), rabbit-anti-GSK-3β, p-GSK-3β, Akt or p-Akt (Cell Signaling). Anti-H3K27me3 (Abcam), anti-SUZ12 (Abcam), anti-EZH2 (Abcam), and rabbit anti-Ring2 (Abcam) anti-flag (Sigma) secondary antibodies were conjugated to FITC or rhodamine (goat anti-rabbit IgG or goat anti-mouse IgG) (Jackson Laboratory) or Peroxidase (goat anti-rabbit IgG or goat anti-mouse IgG) (Amersham Pharmacia Biotech).

Migration assay. Cells infected with vector, Bmi-1, Bmi-1ΔRF mutant, or Bmi-1 RNAi were serum starved for 24 h, then 2×10^4 cells were plated into the upper

chamber of a polycarbonate transwell filter chamber coated with Matrigel (BD) and incubated for 22 hours. Cells inside the chamber were removed with cotton swabs and migratory cells on the lower membrane surface were fixed in 1% paraformaldehyde, stained with hematoxylin and counted (10 random 100X fields per well). Cell counts are expressed as the mean number of cells per field of view. Three independent experiments were performed and the data are presented as the average ± SEM.

Wound healing assay. Cells infected with vector, Bmi-1, Bmi-1ΔRF mutant, or Bmi-1 RNAi were seeded in 6-well plates and grown under permissive conditions until reaching 90% confluence. The cells were then serum starved for 24 h, and a linear wound was created in the confluent monolayer using a pipette tip. Wounds were observed and photographed at various times as indicated in the figure legends. Wound size was measured randomly at five sites perpendicular to the wound. Each experiment was repeated at least three times.

Chromatin immunoprecipitation (ChIP) assays. Briefly, 2×10^6 cells were plated per 100-mm diameter dish and treated with formaldehyde to cross-link chromatin-associated proteins to DNA. The cells were trypsinized and resuspended in lysis buffer, and nuclei were isolated and sonicated to shear the DNA to 500 bp–1 kb fragments (verified by agarose gel electrophoresis). Equal aliquots of chromatin supernatants were subjected to overnight IP with different antibodies as indicated or anti-flag as a negative control. DNA was extracted and the *PTEN* promoter, as well as the first exon, were amplified by PCR. All ChIP assays were performed three to four times and representative results are presented. Sequences of the PCR primers are listed in Supplemental Table 1.

Real-time RT-PCR Analysis. Total RNA from different cell lines and human tissues was extracted using Trizol reagent (Gibco). cDNA was synthesized from 2.5 μg of total RNA using random hexamers. Real-time PCR was carried out using an ABI PRISM 7500 Sequence Detection System (Applied Biosystems). Reactions were run in triplicate in three independent experiments. The geometric mean of the housekeeping gene GAPDH was used as an internal control to normalize the variability in expression levels. The primer sequences are provided in Supplemental Table 2.

Supplemental Table 1. Sequence of primers for ChIP assays

***PTEN*-chip-primer**	**Sequence (5'to3')**
PTEN-1-left	5' aacgtgggagtagacggatg 3'
PTEN-right	5' tagccctcaggaagagacca 3'
PTEN -2-left	5' ggaggcagccgttcggaggattatt 3'
PTEN -2-right	5' ggaaatggctctggacttggcggta 3'
PTEN -3-left	5' ctgaaagggaaggtggaagc 3'
PTEN -3-right	5' ccgtgttggaggcagtagaa 3'
PTEN-4-left	5' ggctcaggcgagggagat 3'
PTEN -4-right	5' gaagaggctgcacggttaga 3'
PTEN- -5-left	5' cactgggcatgctcagtagagcc 3'
PTEN -5-right	5' cataaagagtcccgccacatcacc 3'
PTEN -6-left	5' tctgcgaacgattgtgatccgacag 3'
PTEN -6-right	5' tgagatgggtgcgttgagcagtgtc 3'
PTEN -7-left	5' ccgaggggaaagatgctcgactct 3'
PTEN -7-right	5' cggctcgtttgccctaaaaatgaaa 3'
PTEN -8-left	5' ggctgcagcttcctaccgtt 3'
PTEN -8-right	5'tggtctctgagaaccgagct 3'
PTEN -9-left	5' agctccttttcccacgtttg 3'
PTEN -9-right	5' gaggagtggcaccagtttgg 3'
PTEN -10-left	5' ggttgttaactcctgcacagc 3'
PTEN -10-right	5' agaaagccacaggccattac 3'
p16 -left	5' ggcatcagcaaagtctgagc 3'
p16-chip-right	5' ctgggagacaagagcgaaac 3'
GAPDH-left	5' ggtagggagttcgagaccag 3'
GAPDH-right	5' tcaacgcagttcagttaggc 3'

Supplemental Table 2. Sequence of primers for real-time PCR

Gene	*Forward primer*	Reverse primer	Probe
Bmi-1	*CTGGTTGCCCATTGACAGC*	CAGAAAATGAATGCGAGCCA	FAM-CAGCTCGCTTCAAGATGGCCGC-TAMRA
PTEN	*AGCCGTTCGGAGGATTATTCG*	CTTCTCCTCAGCAGCCAGAG	FAM-ATTCCGCTGCCGCCGCTGCC-TAMRA
E-cadherin	*GAACAGCACGTACACAGCCCT*	GCAGAAGTGTCCCTGTTCCAG	FAM–ATCATAGCTACAGACAATGGTTCTCCAGTTGCT-TAMRA
Snail	*TGCAGGACTCTAATCCAGAGTTTACC*	GTGGGATGGCTGCCAGC	FAM–TCCAGCAGCCCTACGACCAGGCC–TAMRA
GAPDH	*GACTCATGACCACAGTCCATGC*	AGAGGCAGGGATGATGTTCTG	FAM-CATCACTGCCACCCAGAAGACTGTG-TAMRA

SUPPLEMENTAL FIGURE 1

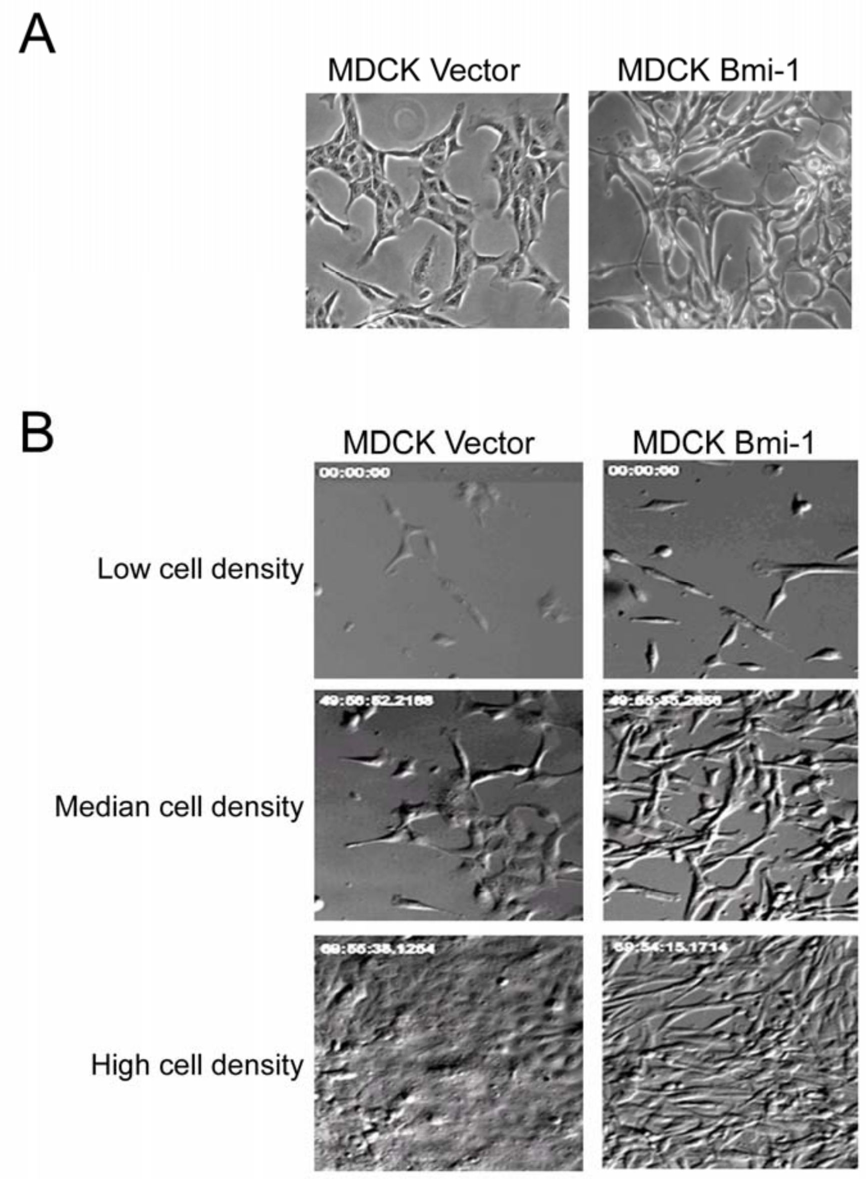

Supplemental Figure 1. Overexpression of Bmi-1 induces EMT in MDCK cells. The cellular morphology of the control MDCK cells and Bmi-1-overexpressed MDCK cells on plastic is seen by phase contrast micrography (A) and by real-time (B). The control MDCK cells (left panel) have cobblestone-like appearances, while Bmi-1-overexpressing MDCK cells (right panel) present a spindle-like, fibroblastic morphology even at different cell densities. Representative real-time images were acquired at the time points shown in the top left corner of each image. Times are shown in hour:min:sec:10-6 sec. Original magnification, 400X.

SUPPLEMENTAL FIGURE 2

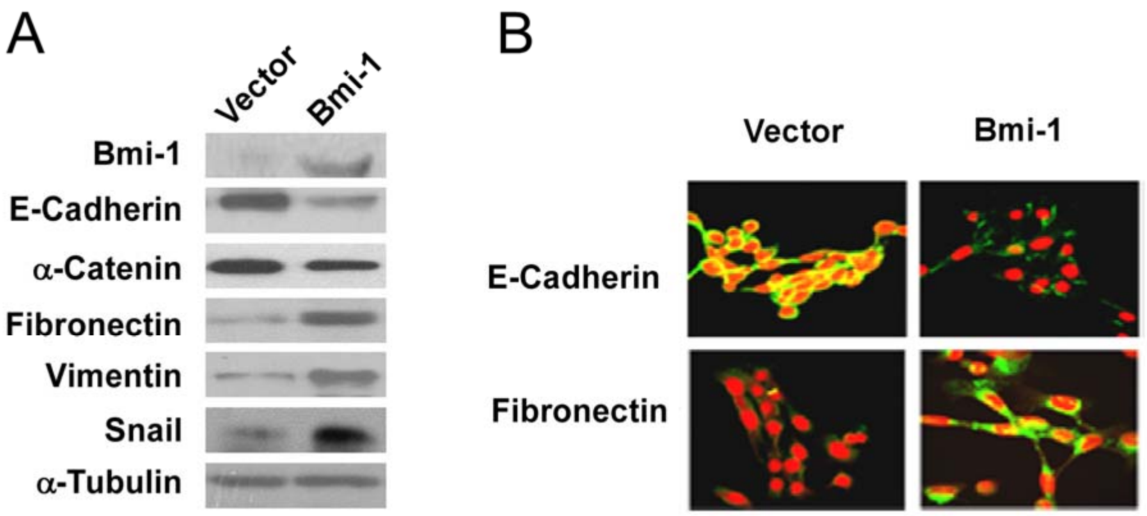

Supplemental Figure 2. Overexpression of the Bmi-1 induces EMT-like molecular alterations in MDCK cells. (A) Expression of Bmi-1, E-cadherin, a-Catenin, Vimentin, and Fibronectin in control and Bmi-1-overexpressing MDCK cells were analyzed by Western blotting. (B) Expression of E-cadherin and Fibronectin in vector and Bmi-1 MDCK cells was analyzed by immunostaining. Original magnification, 400X.

SUPPLEMENTAL FIGURE 3

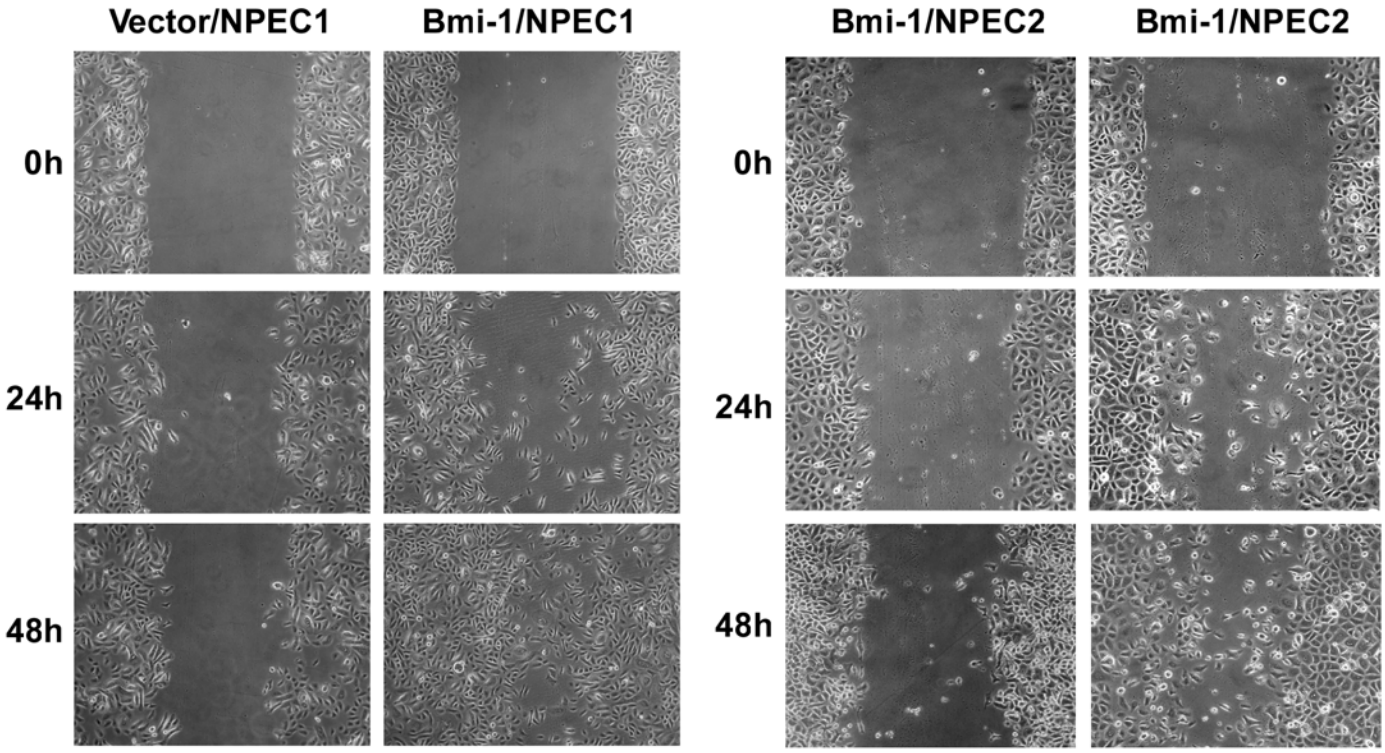

Supplemental Figure 3. Overexpression of the Bmi-1 enhances cell motility in NPECs. A wound was introduced in subconfluent cultures of Bmi-1-overexpressing (Bmi-1/NPEC1, Bmi-1/NPEC2) or control vector cells (as indicated). The rate of wound closure was monitored at different times as indicated. A representative image from three independent experiments is shown. Original magnification, 400X.

SUPPLEMENTAL FIGURE 4

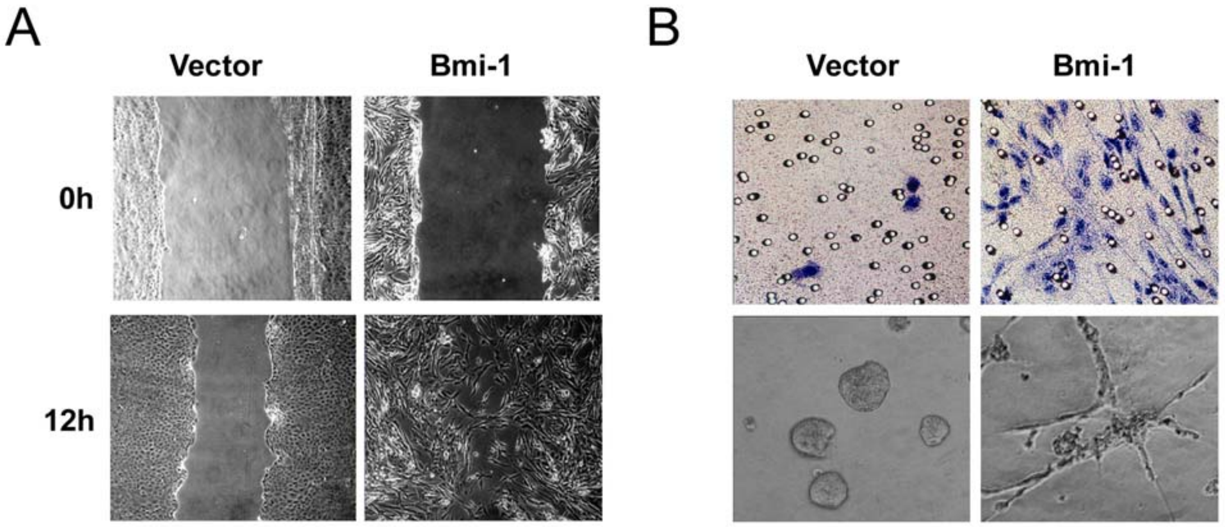

Supplemental Figure 4. The Bmi-1 gene induces a migratory and invasive phenotype in MDCK cells. (A) A wound was introduced on a subconfluent culture of control MDCK cells and Bmi-1-overexpressing MDCK cells, and the rate of wound closure was monitored at 0 and 12 hours. A representative photograph from three independent experiments is shown. Original magnification, 200X. (B) Invasiveness of the cells was analyzed in an invasion assay on a Matrigel coated Boyden chamber. Cells that adhered to the lower surface of the filter were fixed, stained, and visualized (upper). Three-dimensional morphology of MDCK vector and Bmi-1 cells was analyzed after culture in Matrigel (lower). Original magnification, 400X.

SUPPLEMENTAL FIGURE 5

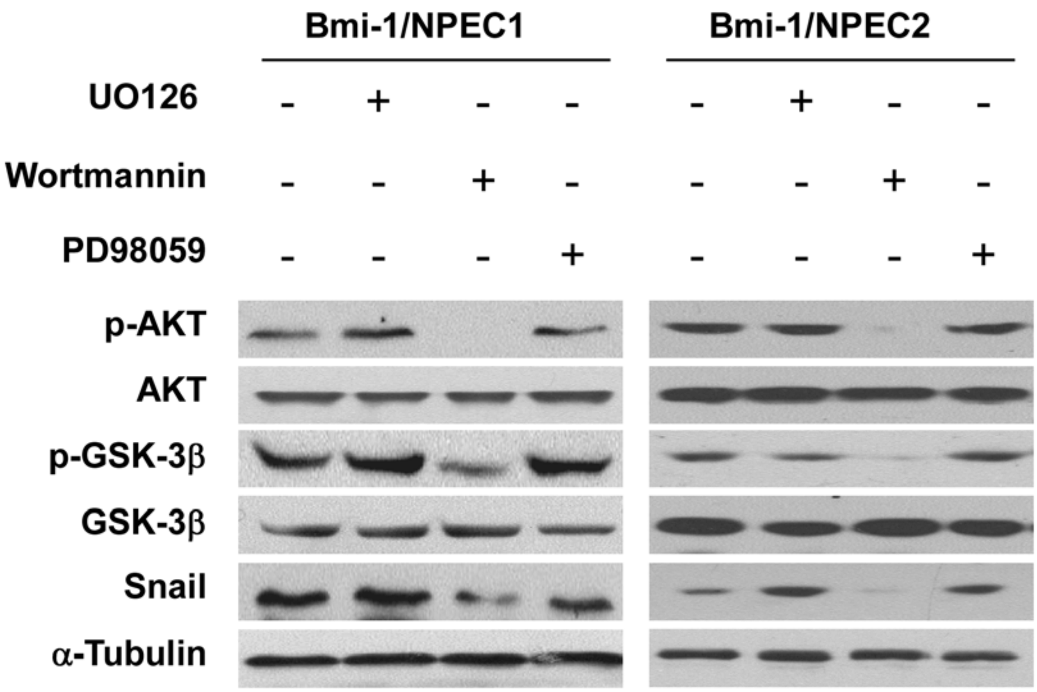

Supplemental Figure 5. Inhibition of PI3K/AKT correlates with downregulation of Snail in Bmi-1/NPECs. Bmi-1/NPECs were pre-treated with either wortmannin (0.1 mM), PD98059 (20 mM), or UO126 (20 mM) for 1h prior to stimulation with EGF (1ng/ml) for 6 h. Cells were then harvested to determine the expression levels of p-Akt, p-GSK-3b, and Snail by Western blot.

SUPPLEMENTAL FIGURE 6

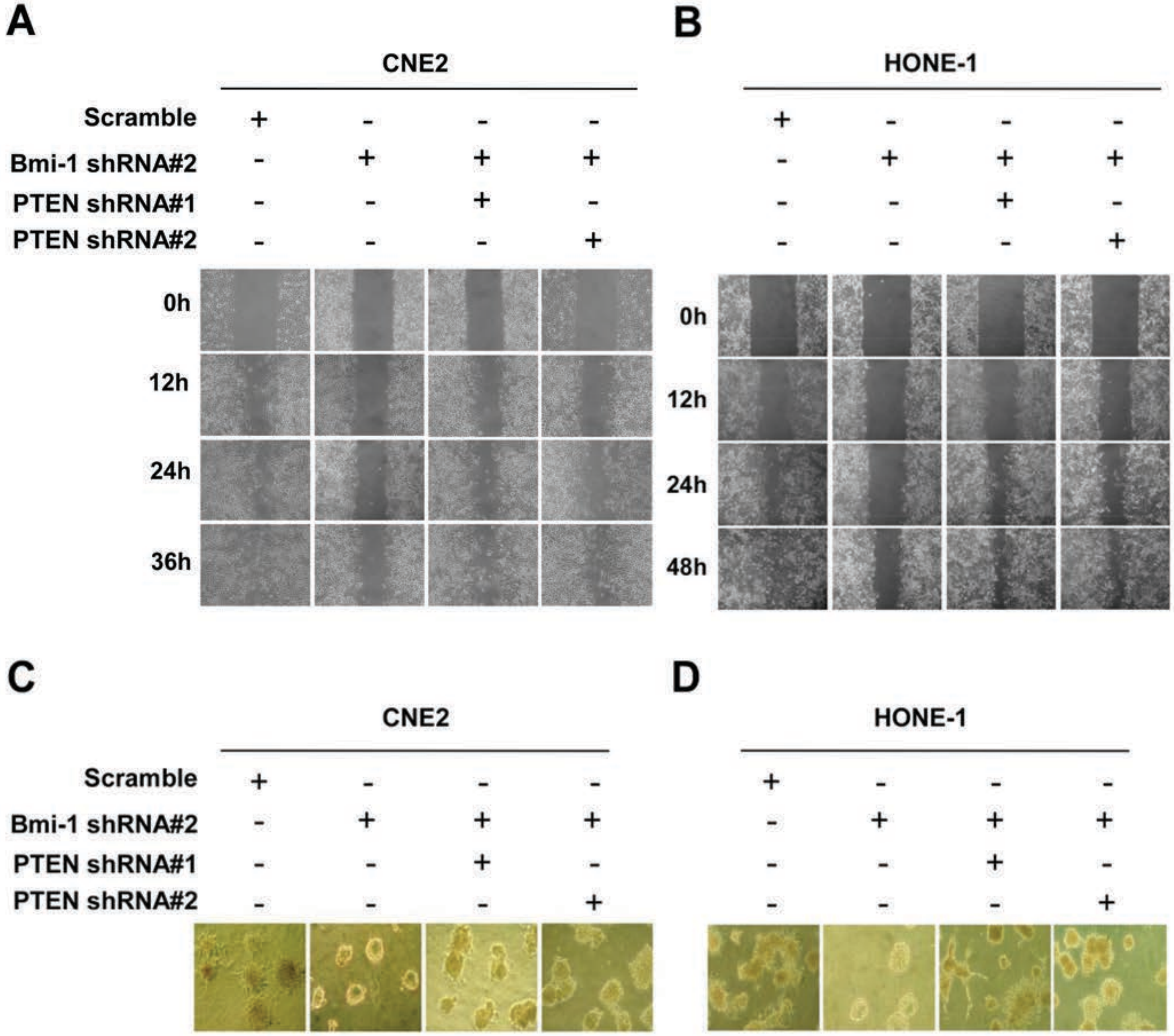

Supplemental Figure 6. Inhibition of PTEN expression by shRNA rescues migratory/invasive phenotype. (A, B) The migratory properties of Bmi-1 knockdown cell lines harboring control shRNA (as indicated) were analyzed. (C,D) Invasiveness of Bmi-1 knockdown cell lines expressing scrambled and PTEN shRNA (as indicated) were analyzed in a Matrigel embedded three-dimensional assay. The morphology of cell colonies in three-dimensional culture were revealed by phase contrast microscopy.

SUPPLEMENTAL FIGURE 7

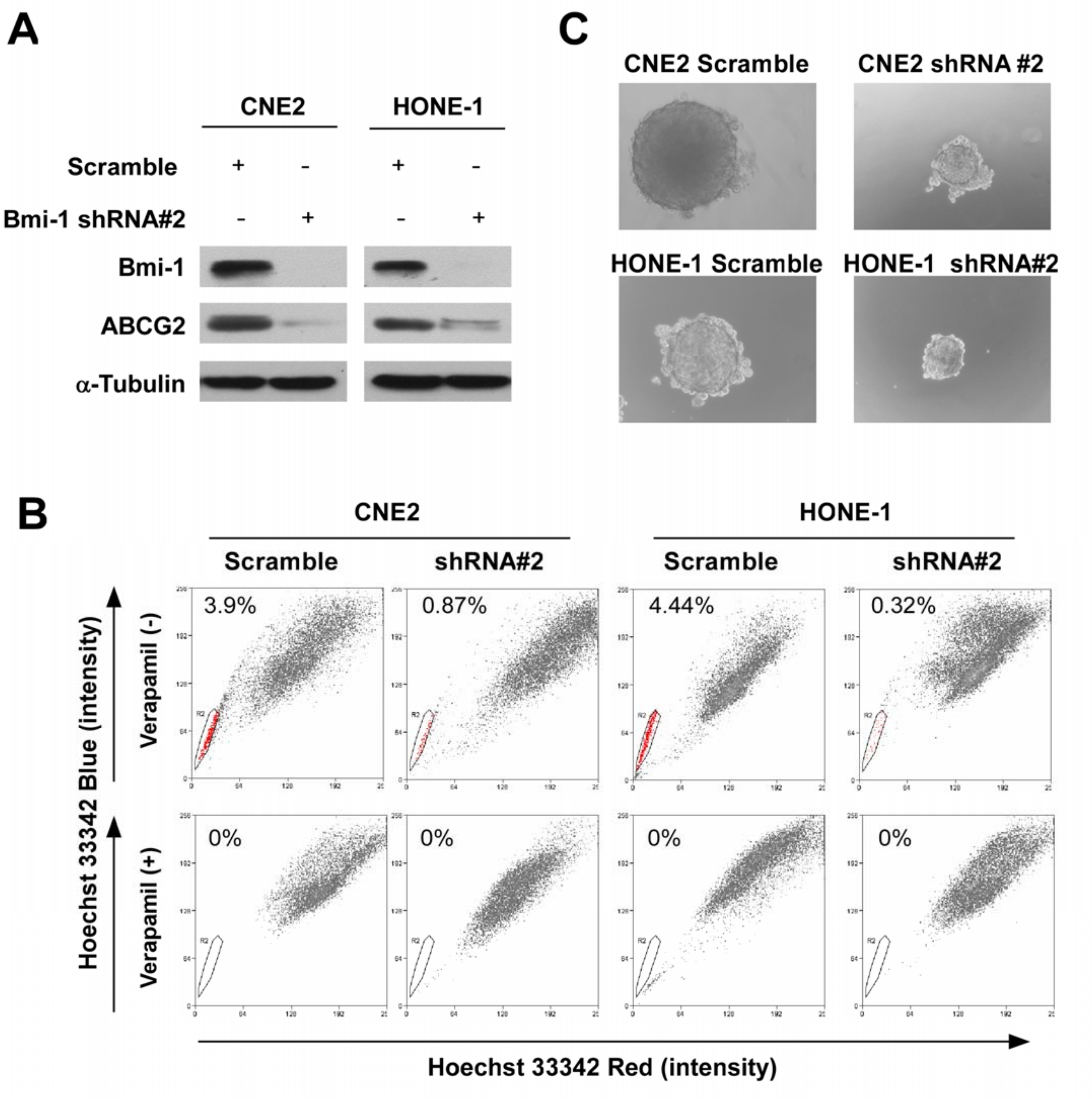

Supplemental Figure 7. Silencing endogenous Bmi-1 expression with small hairpin RNA reduces stem cell phenotype of NPC cell lines CNE2 and HONE-1. (A) The expression of Bmi-1 and the stem cell marker ABCG2 were analyzed by Western blotting. α -Tubulin was used as a loading control. (B) CNE2 and HONE-1 cells with scrambled control shRNA or Bmi-1 shRNA was sorted using Hoechst 33342 stain. The SP proportion was reduced in CNE2 cells (from 3.9% to 0.87%) and in HONE-1 cells (from 4.44% to 0.32%) after silencing of Bmi-1. (C) The stem cell phenotype of control cells and Bmi-1 knockdown cells was observed by culturing in non-adherent, serum-free, sphere forming conditions. Original magnification, 400X.

SUPPLEMENTAL FIGURE 8

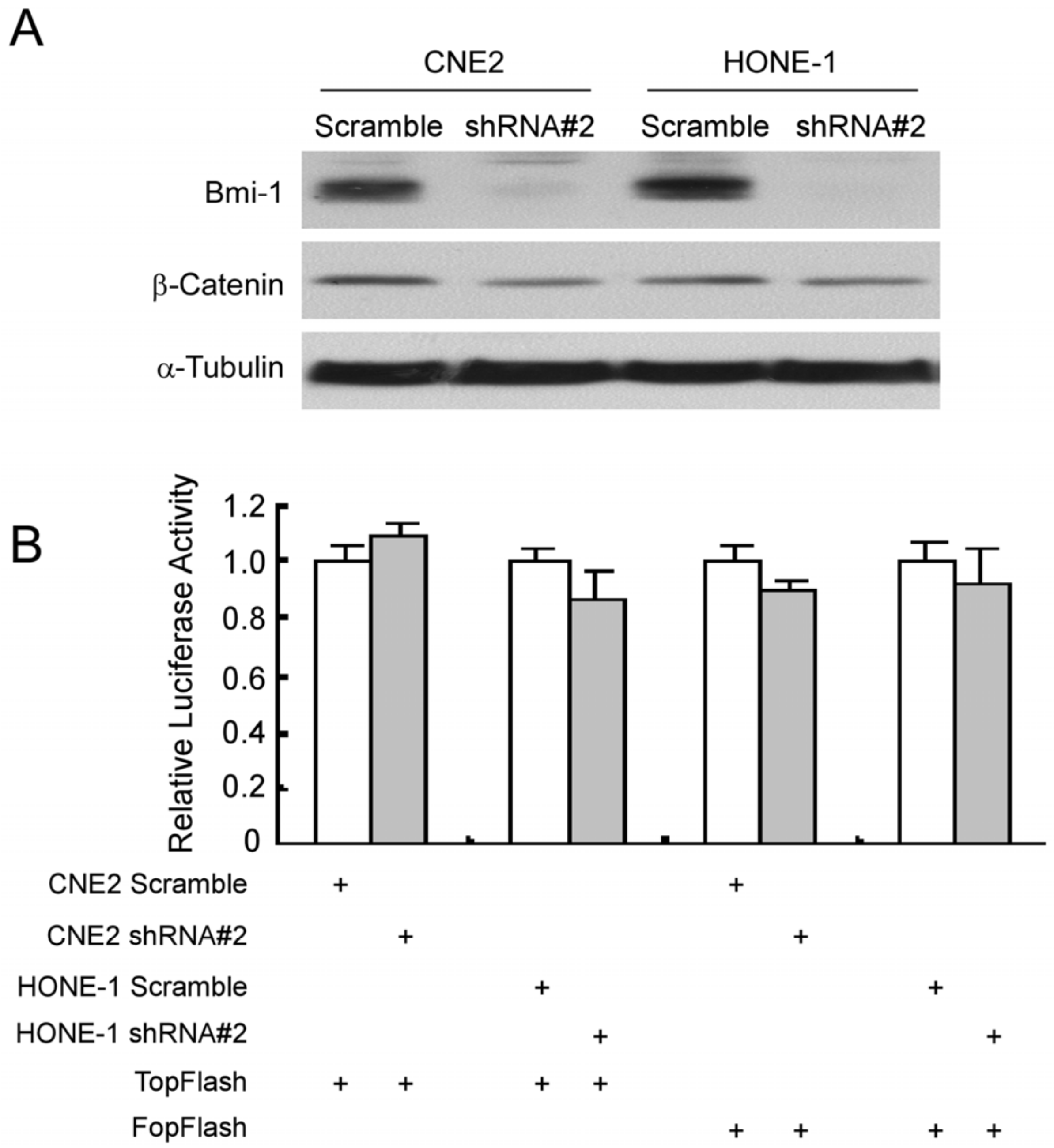

Supplemental Figure 8. Analysis of Wnt/b-Catenin pathway activity. (A) Expression of Bmi-1 and b-Catenin in control and Bmi-1-silenced CNE2 and HONE-1 cells. (B) Transcriptional activity of the Wnt/b-Catenin pathway was measured using the TOP-FLASH Wnt luciferase reporter assay. Neither the TOP-FLASH nor the mutated control reporter FOP-FLASH was affected by the Bmi-1 expression level.

SUPPLEMENTAL FIGURE 9

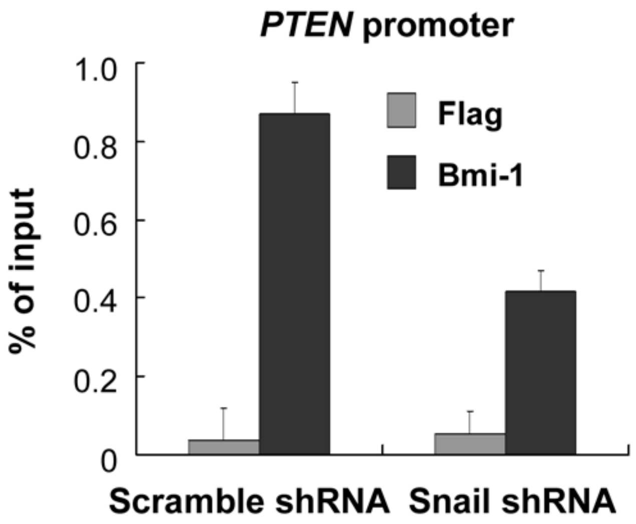

Supplemental Figure 9. Silence of Snail reduces the binding affinity of Bmi-1 to *PTEN* promoter. Binding of Bmi-1 to the *PTEN* promoter was examined by ChIP at varying Snail levels. The result shows the averages ± SEM for two experiments performed in triplicate.

SUPPLEMENTAL FIGURE 10

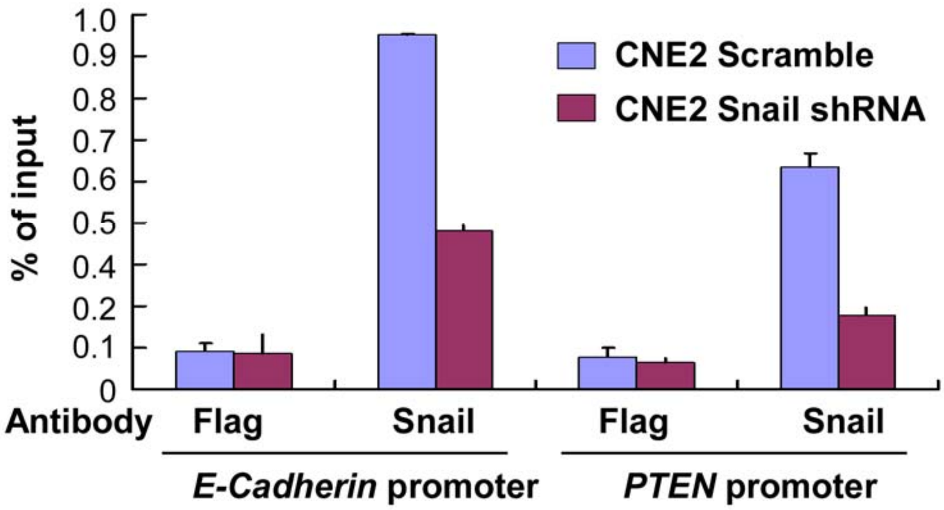

Supplemental Figure 10. Snail is able to bind the *PTEN* promoter. ChIP assays were performed using either anti-Snail antibody or anti-Flag antibody to identify Snail binding affinity for the *PTEN* promoter in CNE2 cells. The E-cadherin promoter was used as a positive control. The results are displayed as the mean ± SEM for two experiments performed in triplicate.

References

1. Song, L.B., Zeng, M.S., Liao, W.T., Zhang, L., Mo, H.Y., Liu, W.L., Shao, J.Y., Wu, Q.L., Li, M.Z., Xia, Y.F., et al. 2006. Bmi-1 is a novel molecular marker of nasopharyngeal carcinoma progression and immortalizes primary human nasopharyngeal epithelial cells. *Cancer Res* 66:6225-6232.
2. Guo, W.J., Datta, S., Band, V., and Dimri, G.P. 2007. Mel-18, a polycomb group protein, regulates cell proliferation and senescence via transcriptional repression of Bmi-1 and c-Myc oncoproteins. *Mol Biol Cell* 18:536-546.
3. Furuta, S., Jiang, X., Gu, B., Cheng, E., Chen, P.L., and Lee, W.H. 2005. Depletion of BRCA1 impairs differentiation but enhances proliferation of mammary epithelial cells. *Proc Natl Acad Sci U S A* 102:9176-9181.
